# Comparative Evaluation on Impacts of Fibronectin, Heparin–Chitosan, and Albumin Coating of Bacterial Nanocellulose Small-Diameter Vascular Grafts on Endothelialization In Vitro

**DOI:** 10.3390/nano11081952

**Published:** 2021-07-29

**Authors:** Max Wacker, Jan Riedel, Heike Walles, Maximilian Scherner, George Awad, Sam Varghese, Sebastian Schürlein, Bernd Garke, Priya Veluswamy, Jens Wippermann, Jörn Hülsmann

**Affiliations:** 1 Department of Cardiothoracic Surgery, University Hospital Magdeburg, 39112 Magdeburg, Germany; jan.n.riedel@icloud.com (J.R.); maximilian.scherner@med.ovgu.de (M.S.); george.awad@med.ovgu.de (G.A.); sam.varghese@med.ovgu.de (S.V.); priya.veluswamy@med.ovgu.de (P.V.); jens.wippermann@med.ovgu.de (J.W.); joern.huelsmann@med.ovgu.de (J.H.); 2 Core Facility Tissue Engineering, Otto-Von-Guericke University Magdeburg, 39106 Magdeburg, Germany; heike.walles@ovgu.de; 3 Department Tissue Engineering and Regenerative Medicine (TERM), University Hospital Würzburg, 97070 Würzburg, Germany; sebastian.schuerlein@gmail.com; 4 Institute of Experimental Physics, Otto-Von-Guericke University Magdeburg, 39106 Magdeburg, Germany; bernd.garke@ovgu.de

**Keywords:** bacterial nanocellulose, small-diameter vascular grafts, endothelialization, tissue engineering, bioreactor

## Abstract

In this study, we contrast the impacts of surface coating bacterial nanocellulose small-diameter vascular grafts (BNC-SDVGs) with human albumin, fibronectin, or heparin–chitosan upon endothelialization with human saphenous vein endothelial cells (VEC) or endothelial progenitor cells (EPC) in vitro. In one scenario, coated grafts were cut into 2D circular patches for static colonization of a defined inner surface area; in another scenario, they were mounted on a customized bioreactor and subsequently perfused for cell seeding. We evaluated the colonization by emerging metabolic activity and the preservation of endothelial functionality by water soluble tetrazolium salts (WST-1), acetylated low-density lipoprotein (AcLDL) uptake assays, and immune fluorescence staining. Uncoated BNC scaffolds served as controls. The fibronectin coating significantly promoted adhesion and growth of VECs and EPCs, while albumin only promoted adhesion of VECs, but here, the cells were functionally impaired as indicated by missing AcLDL uptake. The heparin–chitosan coating led to significantly improved adhesion of EPCs, but not VECs. In summary, both fibronectin and heparin–chitosan coatings could beneficially impact the endothelialization of BNC-SDVGs and might therefore represent promising approaches to help improve the longevity and reduce the thrombogenicity of BNC-SDVGs in the future.

## 1. Introduction

Coronary artery bypass grafting (CABG) is the most frequently performed heart surgeries in the western world. In Germany and the USA alone, more than 450,000 CABG operations are conducted every year, using small caliber vascular grafts with an inner diameter of 6 mm or below to redirect the blood distal to the blockage [1,2]. Autologous vessels—especially the internal thoracic artery, the radial artery, or the saphenous vein—are considered the gold standard for graft material in CABG surgery, as they represent the best compromise between availability and long-term patency rate, which is mainly dependent on the biological function of the grafts, e.g., the endothelial integrity and mechanical characteristics of the vessel wall [3,4]. While the optimal bypass graft has not yet been found, access to autologous vessels that are expected to have a long-term patency of over 90%, such as the internal thoracic artery, is often limited due to vascular diseases, pre-surgery, or increased risk of wound infections [5,6,7], and alternatives, such as the saphenous vein, are expected to occlude in over 50% of the cases after 10 years [8]. Therefore, alternatives are warranted, and today, tissue-engineered vascular grafts (TEVG) are thought to be a promising approach in the development of synthetic grafts mimicking the biological functionality of autologous vessels with unlimited availability.

Despite many different approaches that have been examined to date, none have been able to show satisfactory long-term patency in application-oriented experimental setups [9]. Common causes of low patency rates, such as intimal hyperplasia and thrombus formation, could be addressed with a functional endothelium [10,11]. However, the time-consuming fabrication of in vitro endothelialized grafts does not appear to be appropriate, as current guidelines recommend revascularization within a few days to a few weeks following diagnosis [12]. Therefore, acellular grafts for off-the-shelf use that become endothelialized in situ after implantation are warranted. Among the variety of different approaches in the development of small-diameter TEVGs, acellular scaffolds made of synthetic polymers are of particular interest, because they are reproducible, possess satisfying mechanical properties, and are capable of being stored for longer periods without the need for long-term precultivation and cell seeding [13].

We developed an acellular TEVG composed of bacterial nanocellulose (BNC) with a small inner diameter (below 5.00 mm). The BNC grafts have shown satisfactory short-term results, which were characterized by good surgical manageability and burst pressure in the physiological environment. These grafts were also successfully surface modified to reduce the thrombogenicity, but the long-term results are still limited as reflected by low patency rates and lack of sufficient endothelialization [14,15,16,17].

The in vivo endothelialization of acellular scaffolds after implantation works by two different mechanisms: (i) continuous ingrowth of vascular endothelial cells (VECs) from the anastomotic region and (ii) attachment of circulating endothelial progenitor cells (EPCs) directly from the blood [18]. The homing and adhesion of VECs and EPCs is mainly dependent on expression of proteins on the respective cell surface; in fact, surface coating with substances that resemble the function of these proteins has shown promising results with regard to adhesion of endothelial cells [18,19].

With regard to BNC grafts, coatings with fibronectin, heparin–chitosan, or albumin are interesting approaches for future surface modification of BNC grafts. Fibronectin, of note, is a protein of the extracellular matrix modulating the adhesion and proliferation of endothelial cells in vivo [20]. It has been shown that fibronectin coating led to significant improvements with endothelial cell expansion on the BNC surface [21]. Crosslinking of BNC with heparin led to increased endothelialization and reduced activation of the coagulation cascades [22,23], and heparin coating could be of further interest with respect to intimal hyperplasia, as it is reported to inhibit the growth of fibroblasts [24]. This is particularly appealing, as fibroblast growth can result in intimal hyperplasia and thus late occlusion of the synthetic grafts. Chitosan, a polysaccharide derived from the chitin shell of crustaceans with antibacterial and anticoagulative properties, has recently shown excellent results with regard to endothelial cell adhesion and proliferation when used as a component of small-diameter vascular grafts [25,26]. A combination of heparin and chitosan has already been introduced as a coating for BNC based grafts but was only examined with non-human endothelial cells [22]. This encouraged us to test this combination in a more clinical setup using human VECs. Additionally, albumin also seems to be a promising coating to induce EPC homing for an enhanced in situ self-endothelialization on vascular devices [27].

However, more information to verify these beneficial impacts for clinically relevant cells by proof of principle studies in a dynamic environment is still warranted to help the design and further development of TEVGs, as many studies investigating the effect on the above mentioned coatings on endothelialization have been conducted using non-human cells or human umbilical vein endothelial cells [21,23,28,29], which differ significantly in function and receptor expression from human saphenous vein endothelial cells or EPCs [30,31], and which therefore relativize the translational approach of these studies. This translational approach is further limited when in vitro experiments do not include medium perfusion [21,22,32,33,34], because the activity of integrins that mediate cell adhesion is dependent on shear stress and is regulated accordingly [35]. With this comparative study, we report the endothelialization of small-diameter vascular grafts made of BNC which were either coated with fibronectin, heparin–chitosan, or albumin in a clinically relevant in vitro setting and tried to simulate the natural and necessary stimulation by wall shear stress in a bioreactor.

Upon cell seeding with human primary EPCs or human saphenous vein endothelial cells, these grafts were cultivated under both static and dynamic conditions, using an in vitro approach and a bioreactor perfusion system. Besides proliferation assays, the endothelialized grafts were analyzed histologically with regard to the extent of endothelialization and endothelial cell characterization, including their functionality. Thus, using the current state-of-the-art equipment, we hope to better screen for the potential for translational applications.

## 2. Materials and Methods

All procedures were in accordance with the ethical standards of the responsible committee on human experimentation of the University Hospital of Magdeburg (application number 81/18) and with the Helsinki Declaration of 1975, as revised in 2000. Informed consent was obtained from all participants for being included in the study.

### 2.1. BNC Graft Production

The BNC grafts with an inner diameter of 5.0 mm were produced in a standardized fashion as described before [36,37,38]. Briefly, in a customized bioreactor, cylindrical templates made of bamboo with a diameter of 5.0 mm were moved periodically between air space and a reservoir, which was filled with liquid culture medium and bacteria of the genus Komagataeibacter xylinus (German Collection of Microorganisms and Cell Cultures GmbH, DSM 32384). During dipping, the template was loaded with culture medium and bacteria, and after leaving the liquid, BNC formation took place on the template surface. After the cultivation time, the templates were removed from the BNC, leaving a vascular graft with an inner diameter that corresponded to the bamboo template. The BNC grafts then underwent several post-processing steps, including washing in ultrapure water and boiling in sodium hydroxide solution, followed by autoclaving.

### 2.2. Coating Procedures

**Figure 1:**
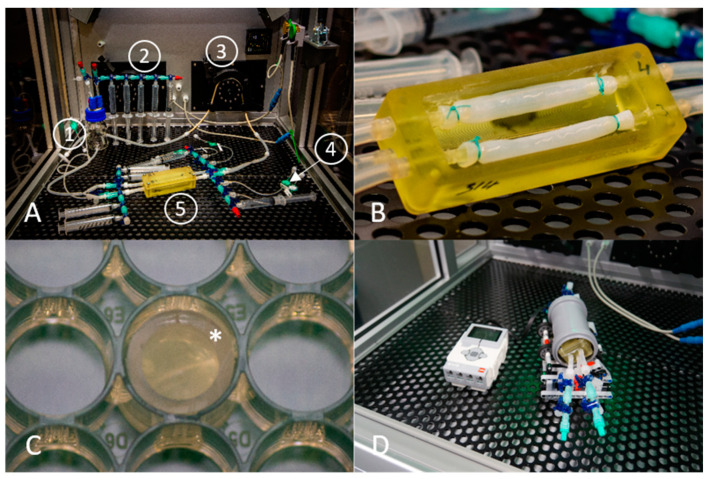
Laboratory setup for dynamic and static endothelialization experiments: (**A**) Perfusion system which was set up in an incubation chamber with controlled atmosphere (5% CO_2_). (1) Medium reservoir, (2) Syringes for media sampling, (3) Roller pump, (4) 0.2 µm sterile filter for adding WST-1 cell proliferation assay reagent, (5) 3D printed reactor containing the BNC grafts. (**B**) Enlarged view of the BNC grafts mounted on the 3D printed bioreactor, secured with surgical sutures. (**C**) A piece auf autoclaved PVC tube (*) was added on top of the patch and placed in a 48-well plate to avoid cells running off the BNC patch during the first 24 h of static cultivation. (**D**) Perfusion chamber mounted to a self-made rotation unit. Left: Control unit. Right: The bioreactor was positioned inside a commercially available piece of drain pipe and rotated on the rotation unit to promote cell distribution over the whole BNC graft surface.

To investigate the effect of surface coating on the in vitro endothelialization, three different coatings (human fibronectin, heparin–chitosan, and human albumin) were examined and compared to uncoated BNC grafts. Thus, the different coated BNC grafts are referred to as fibronectin, heparin, albumin, or uncoated group. Static culture was performed in 48-well plates, where round patches of 10 mm diameter were punched out from the tubular, uncoated grafts with a medical biopsy punch and were subsequently coated. For dynamic culture, the coating was performed directly on the tubular graft. All coating procedures for the patches and the tubular BNC scaffolds in the heparin group were performed in a beaker glass. For fibronectin and albumin coating of the tubular BNC scaffolds, autoclaved BNC grafts were mounted to the custom-made 3D printed bioreactors and fixed with non-absorbable surgical sutures under sterile conditions as shown in Figure 1. The coating procedure for the grafts was then performed by adding the coating solutions with syringes directly to the mounted grafts through the luer-lock adapters.

#### 2.2.1. Heparin–Chitosan Coating

The heparin–chitosan coating on BNC was done in a modified way than originally described in 2017 by Li et al. [22]. Briefly, the BNC constructs were immersed in an aqueous chitosan solution containing 1% (*v*/*v*) acetic acid (Carl Roth, Karlsruhe, Germany), 1% (*v*/*v*) glycerin (Sigma-Aldrich, St. Louis, MI, USA) and 1 mg/mL chitosan (molecular weight 600,000 to 800,000 Da, ≥90% deacetylated; VWR Chemicals, Radnor, PA, USA) for 24 h under agitation at room temperature (RT), followed by fixation in 0.1 M NaOH aqueous solution (Sigma-Aldrich, St. Louis, MI, USA) for 24 h without agitation. After extensive washing with 1% (*v*/*v*) penicillin/streptomycin (pen-strep) in distilled water (ThermoFisher Scientific, Waltham, MA, USA), the BNC constructs were directly transferred into a glass beaker containing 0.05 M MES buffer (Sigma-Aldrich, St. Louis, MI, USA) for 2 h. Then, the BNC scaffolds were coated with heparin by immersion in an EDC/NHS crosslinking solution for the formation of amide and ester bonds, prepared with 0.05 M MES buffer, containing 1 mg/mL heparin, 0.25 M NaCl, 0.03 M NHS, and 0.06 M EDC (all purchased from Sigma-Aldrich, St. Louis, MI, USA) for 24 h in a water bath shaker at 30 °C. After extensive washing with pen-strep solution for 2 h, the tubes were stored in pen-strep solution, sterilized by gamma irradiation with 13 Gray, and stored at 4 °C.

#### 2.2.2. Albumin and Fibronectin Coating

The coating procedure was modified from the procedure published by Kuzmenko et al. [21]. The 1-cyano-4-dimethylamino pyridinium tetrafluoroborate (CDAP), acetonitrile, triethylamine sodium acetate, human fibronectin, and human albumin were purchased from Sigma-Aldrich (St. Louis, MI, USA). First, the BNC surface was activated with CDAP as the respective crosslinker, generating a cyanate ester derivate. For this, a CDAP crosslink solution was prepared by dissolving 50 mg CDAP in 1 mL acetonitrile. For activation, the required amount of CDAP per cm^2^ patch surface for the inner graft surface was ensured by setting the concentration to 50 µmol/cm^2^ in an excess of volume, ensuring that the patches were completely immersed or the tubes were completely filled with CDAP crosslink solution. After adding the CDAP crosslink solution to the BNC grafts or patches, they were incubated for 30 s at room temperature. Then, the same volume of 0.2 M triethylamine (TEA) solution prepared in distilled water was added and incubated for 2 min, followed by discarding the CDAP/TEA solution. After rinsing the BNC patches/grafts with 0.1 M sodium acetate buffer (pH 3.0), prepared in distilled water, the BNC patches/grafts were extensively rinsed with 1% pen-strep solution until the pH was neutralized.

For subsequent bioconjugation with the respective protein by an isourea bond to the amino acids of the protein, an aqueous solution containing 100 µg/mL human fibronectin or albumin was added to the CDAP activated BNC patches/grafts, ensuring that the patches were completely covered or no air remained in the tubular grafts. After incubation for 12 h at 37 °C, the patches and grafts were rinsed with phosphate buffered saline solution containing 1% (*v*/*v*) pen-strep, gamma irradiated (13 Gray for patches and 25 Gray for tubular grafts), and stored at 4 °C.

### 2.3. Coating Analyses for Quality Control

#### 2.3.1. XPS Analyses

X-ray photoelectron spectroscopy (XPS) was used to analyze the surface chemistry of the coated BNC grafts [39]. Therefore, rectangular samples (0.5 × 0.5 cm) were cut from coated BNC grafts and freeze dried. Then, the samples were analyzed by XPS using a PHI 5600 ESCA System (Physical Electronics Inc., Division of ULVAC-PHI, 18,725 Lake Drive East in Chanhassen, MN 55317, USA), equipped with a Spherical Capacitor electron energy Analyzer (energy resolution 25 meV, PHI 5600 ESCA System) and a Dual Anode X-ray Source operating with Mg K-alpha at 14 kV and 400 W. The analyzed lateral area had a diameter of 800 µm, using a detection angle (Source to SCA) of 54°. To define the atomic concentration of the analyzed elements, multiplex spectra were measured for each sample.

#### 2.3.2. Heparin Detection

To verify sufficient heparin presence at the graft surface, we used a toluidine blue staining modified from the one described by Hinrichs et al. [40]. Samples of coated and uncoated BNC grafts were stained in a 0.04% toluidine blue (Sigma-Aldrich, St. Louis, MI, USA) staining solution prepared in 0.01 M HCL containing 2 mg/mL NaCl for 12 h, followed by extensive washing with distilled water and incubation for 24 h in distilled water before images were obtained.

#### 2.3.3. Albumin and Fibronectin Detection

To validate the presence of the respective functional proteins at the luminal surface after coating, grafts were sampled and subjected to immunofluorescent evaluation. Therefore, 0.5 cm long sections from exemplary tubular grafts coated with albumin or fibronectin were frozen in compound (Tissue-Tek O.C.T. compound, Sakura Finetek Europe B.V., Alphen aan den Rijn, NL) and thereafter sectioned using a Leica CM 1950 cryostat (Leica Biosystems, Nussloch, Germany). Recombinant monoclonal rabbit anti-human serum albumin antibody (MA5-29022, ThermoFisher Scientific, Waltham, MA, USA) and recombinant monoclonal rabbit anti-human fibronectin antibody (MA5-32509, ThermoFisher Scientific, Waltham, MA, USA) were used as the primary antibody and donkey anti-rabbit Alexa Fluro 488 (Jackson ImmunoResearch, West Grove, PA, USA) as the secondary antibody. Upon fixation in 4% paraformaldehyde (PFA), the graft slices were blocked with 3% standard donkey serum for 30 min and then incubated with the primary antibodies at a dilution of 1:50 in 3% donkey serum overnight. After washing, the fluorescent dye-conjugated secondary antibody at a dilution of 1:500 in 3% donkey serum was incubated on the slice in the dark for 1 h. After mounting, the slices were analyzed using the EVOS Auto 2 (ThermoFisher Scientific, Waltham, MA, USA).

### 2.4. Cell Isolation and Cell Culture

#### 2.4.1. Human Saphenous Vein Endothelial Cells (VECs)

The isolation of endothelial cells from human saphenous veins for use in vascular prostheses seeding experiments is a standard method and was described in 1984 by Watkins et al. [41]. For cell isolation, the vein sections obtained from patients undergoing coronary artery bypass surgery were flushed with PBS to remove blood and thereafter filled with 0.4% Collagenase A solution (Sigma-Aldrich, St. Louis, MI, USA) and incubated at 37 °C for 30 min. By rinsing with PBS, the cell suspension was collected and plated as passage 0 on 0.1% gelatin-coated cell culture flasks using a cell specific growth medium (Endothelial Cell Growth Medium C22110, Promocell, Heidelberg, Germany) containing 1% pen-strep solution and 10% fetal calf serum (FCS) with a density of 0.6–1.0 × 10^5^ cells/cm^2^. For long-term stand cell culture, the cells were passaged at 90% confluence using 0.05% trypsin/EDTA (ThermoFisher Scientific, Waltham, MA, USA). Also, for the standard growth medium, the FCS content was reduced to 5% for passage 1 and 2% for later passages. For seeding experiments, endothelial cell cultures were used until passage 5 as a maximum. Cells were stored at −150 °C (50% FCS, 10% DMSO).

#### 2.4.2. Human Endothelial Progenitor Cells (EPCs)

Human endothelial progenitor cells were isolated from peripheral blood obtained from healthy volunteers. The procedure was carried out as described by Ormiston et al. [42]. Briefly, 60 mL of fresh blood was drawn in sodium citrate tubes (Vacutainer 367704, Becton Dickinson [BD] GmbH, Heidelberg, Germany), and peripheral blood mononuclear cells (PBMCs) were obtained by density gradient centrifugation (Ficoll-Paque Plus, GE Healthcare, IL, USA). After washing with PBS, the PBMCs were resuspended in endothelial cell medium (Endothelial Growth Medium-2MV, CC-3202, Lonza, Basel, Switzerland) with 10% FCS content and plated in T75 flasks coated with 5 µg/cm^2^ collagen (Type 1 Collagen, derived from rat tail, 35-4236; BD Biosciences, Heidelberg, Germany).

According to the protocol, the cells were passaged for the first time after initial outgrowth colonies reached a size of approximately 1000–2000 cells/colony, counted on microscopy images. Deviating from the protocol of Ormiston et al., TrypLE (ThermoFisher Scientific, Waltham, MA, USA) was used for cell dissociation. The cells were cultured in uncoated TPP tissue culture flasks (Techno Plastic Products AG, Trasadingen, Switzerland) after passage 5 with an FCS content of 5% for long-term cell culture. The cells were passaged at 90% confluence, cultivated to a maximum of nine passages and stored at −150 °C (50% FCS, 10% DMSO).

### 2.5. Cell Characterization

A detailed description of endothelial cell characterization is described in the Supplementary Material (Figures S1 and S2).

#### 2.5.1. Phase Contrast Microscopy

For morphologic characterization, cells were observed daily with a conventional phase contrast microscope during culture (EVOS XL Core Imaging System, ThermoFisher Scientific, Waltham, MA, USA). In the case of apparent fibroblast contamination, those cultures were not used for experiments.

#### 2.5.2. AcLDL Assay

To exclude potential degeneration of the endothelial cells, we actively tested the endothelial functionality by the ability for the uptake of acetylated low density lipoproteins by the scavenger cell pathway using a commercial assay (Alexa Fluor 488 AcLDL assay, ThermoFisher Scientific, Waltham, MA, USA) [43,44,45]. For AcLDL assay, the endothelial cells were seeded in 24-well plates and cultivated until 90% confluence. The assay was performed as recommended by the manufacturer. Briefly, the cells were washed two times in a 1% bovine serum albumin (BSA)/PBS^+^ solution for 5 min, followed by adding 400 µL of cell culture medium containing 0.0.125% AcLDL assay solution and incubation at 37 °C for 3.5 h. After adding one drop of NucBlue live staining solution for Deoxyribonucleic acid (DNA) staining, (ThermoFisher Scientific, Waltham, MA, USA), the cells were incubated for another 30 min in the incubator. After washing another three times, images were directly obtained with a conventional fluorescent microscope (EVOS FL Auto 2 cell imaging system, ThermoFisher Scientific, Waltham, MA, USA).

#### 2.5.3. Flow Cytometry

The isolated endothelial cells were characterized using flow cytometry (FC) for the expression of the endothelial cell markers von Willebrand Factor, CD 144, CD31, and CD34. A detailed description of the methodology and the results is given in the supplementary files.

### 2.6. Control Based Construct Culture

To get a general impression of the cell growth characteristics on the corresponding BNC surfaces, we chose a static model of cultivation in 48-well plates, and to achieve further understanding of the cell adhesion property in a so-called physiological environment, we cultivated the cells under media perfusion. For both dynamic and static conditions, a control based approach was chosen, where cells were seeded in the same density on 0.1% gelatin-coated well plates and used for normalization of metabolic activity, where values from control cells were set to 100% as optimal cell adherence to the cell culture flask bottom and respective proliferation was considered. To determine optimal cell densities for seeding, previous examinations were conducted where cells were seeded on uncoated BNC patches and gelatin-coated control well plates. After static incubation for 24 h and DNA staining, the cell distribution was examined with immunofluorescence microscopy, determining the lowest cell concentration that avoids overly dense growth in the control wells but still reaches a seeding efficacy of over 5%, as reported in the literature for endothelialization-based cell seeding experiments [46]. Based on these results, the seeding density was set to 5 × 10^4^ cells per cm^2^ surface area. A concentration of 10% FCS in the respective cell culture medium was chosen to provide optimal nutrients to the endothelial cells. 2 × 10^6^ cells from each experiment were frozen and stored at −150 °C for subsequent cell characterization (50% FCS, 10% Dimethylsulfoxid (DMSO)).

#### 2.6.1. Static Culture Experiments

The coated and uncoated patches were placed in a 48-well plate with the luminal side facing upwards. The position inside the well was fixed by placing a piece of autoclaved polyvinylchloride (PVC) tube (inner diameter of 8 mm, wall thickness of 1 mm) inside the well (Figure 1), thereby ensuring that cells did not run off the BNC patch surface. Either VECs (*n* = 10 for each coating) or EPCs (*n* = 5 for each coating) were thawed and cultured for 4 days in T75 flasks on 0.1% gelatin coating. After harvesting, the patches were subjected to passive seeding and the cells were allowed to settle. After 24 h cultivation for cell settling and adhesion, the PVC tubes were removed and the patches were placed into new wells for subsequent cultivation for 96 h. The media was changed every day.

#### 2.6.2. Perfusion Culture Experiments

##### Bioreactor Perfusion System

To study the behavior of the seeded endothelial cells on the grafts under perfusion, we used customized 3D printed bioreactors and a perfusion platform that was described by Schuerlein et al. [47], shown in Figure 1A. The 3D printed bioreactors allowed us to perfuse two BNC tubes connected to one pump system simultaneously (Figure 1B). All parts of the reactor were autoclavable and the BNC grafts were attached to the connectors with surgical sutures. To exclude possible bias from different lengths of the silicone tubes, the volume inside the perfusion path between the two stop cocks upstream and downstream to the BNC tube was determined individually for each perfusion slot. The perfusion system further consisted of silicon tubes and a reservoir bottle as shown in Figure 1A. After autoclaving, the system was assembled under the cell culture bench, and syringes for medium sampling as well as 0.2 µm sterile filters (Intrapur Neonat infusion filter, B. Braun Melsungen AG, Germany) for the cell proliferation assay were attached before the closed perfusion system was mounted on the perfusion platform. The perfusion platform consisted of an incubator with a controlled atmosphere (5% CO_2_) and temperature (37 °C) and a roller pump for medium perfusion.

##### Seeding Technique

The respective endothelial cells were thawed and cultured 4 days in advance in T75 flasks with 0.1% gelatin coating for VECs or no coating for EPCs and 10% FCS. On the first day of the experiment, the cells were harvested after setting the cell concentration; the suspension was taken up in a 10 mL syringe. Then the cell suspension was manually perfused through the perfusion path that integrates the graft in between two three-way stopcocks, holding the bioreactor in an upright position, until the whole graft was filled with the cell suspension, and the connectors were closed with sterile caps. Cell settling and adhesion were supported by incubation on a custom-made roller shaker (Figure 1D) at a speed of 0.05 rpm at 37 °C for 30 min, followed by static incubation for 3.5 h at 37 °C. After incubation, the bioreactors were connected to the tubing system as described before and mounted on the perfusion platform. The perfusion pump was set to pulsatile flow and a medium flow rate of 1.5 mL/min. The perfusion system was filled with a total of 50 mL of medium and samples were taken every day for analytical evaluation. After 96 h, the perfusion was stopped and the BNC grafts were removed, divided into equal parts, and stored in PBS for subsequent analyses. Cells seeded on 0.1% gelatin-coated (VEC) or uncoated (EPC) 12-well plates with the same cell number were used as controls. For each coating group, *n* = 4 BNC grafts were cultured and analyzed.

### 2.7. Metabolic Activity (WST-1 Assay) of Cells on BNC Constructs, Normalization

A WST-1 ((4-(3-4-iodophenyl)-2-(4-nitrophenyl)-2H-5-tetrazolio)-1,3-benzenedisulfonate) proliferation assay (Sigma-Aldrich, St. Louis, MI, USA) was used to determine the overall cell viability and proliferation of the cells. For the assay, a working solution was prepared by diluting the WST-1 reagent in the respective cell culture medium to a final concentration (*v*/*v*) of 12.5% in growth medium. The WST-1 cell proliferation assay was conducted under both static and dynamic conditions and for both cell lines, VEC and EPC.

#### 2.7.1. Static Cultures

The WST-1 cell proliferation assay was performed after 24 h and 96 h of cultivation. 500 µL of the WST-1 working solution was added to the respective wells after washing with PBS for an incubation time of 4 h at 37 °C.

#### 2.7.2. Perfusion Cultures

Consistent with the static cultivations, WST-1 proliferation assay was performed after 24 h and 96. The perfusion was stopped and 3 mL of WST-1 working solution was added to the lumen of each BNC graft individually via the connected 0.2 µm filters (Intrapur Neonat infusion filter, B. Braun Melsungen AG, Germany) to avoid contamination. After 4 h of incubation, the WST-1 working solution was collected and the perfusion was re-established. In parallel, the positive controls in the 12-well plate were incubated in 437 µL of WST-1 working solution, which corresponded to the same amount of WST-1 volume per seeded cells per surface area.

The extinction of the WST-1 working solution was determined for each sample with a multiplate reader (Infinite 200 pro M Plex, Tecan Trading AG, Switzerland) at 440 nm (reference wavelength 600 nm) and blanked to working solution. All samples were measured as duplicates. For dynamic conditions, the measured absorbance had to be multiplied with a dilution factor, depending on the luminal volume in the perfusion path. The values for WST-1 proliferation index obtained after 24 h and 96 h were normalized to controls as described before.

### 2.8. AcLDL Assay on Seeded BNC Cell Constructs

The AcLDL assay was performed as described above with light modifications. After 96 h of cultivation, the seeded BNC constructs (patches or sections from tubular BNC grafts) were immersed in 2 mL Eppendorf tubes filled with PBS for washing. After 5 min of incubation, the samples were transferred to a new micro-reaction tube with fresh PBS. The washing step was repeated three times. Afterwards, the AcLDL assay was performed as described before with an excess of medium containing 0.0125% AcLDL assay solution. Following the incubation time, the samples were washed again in PBS and images were acquired with a fluorescence microscope (EVOS FL Auto 2 cell imaging system, ThermoFisher Scientific, Waltham, MA, USA). Subsequently, the samples were stored in PBS at 4 °C until they were additionally stained with rhodamine-conjugated phalloidin dye as described above. Finally, further images were taken with a Leica confocal imaging system (Leica TCS SP8, Leica Microsystems GmbH, Wetzlar, Germany).

### 2.9. Cryosectioning and Immunofluorescence of BNC Constructs

Acridine Orange staining, a fluorescent dye that reports both cytoplasmic RNA and DNA in the nuclei, was used to get a visual impression of cell survival, cell morphology, and its distribution on the BNC surface [48,49]. To determine the shape of the cytoskeleton of the endothelial cells, we additionally stained for F-actin, which is a commonly accepted method for morphological studies on endothelial cells [50,51]. Furthermore, we complemented a CD-31 staining to detect preserved endothelial character of the cells [50]. For cultivated constructs, cell-specific markers were evaluated after fixation in 4% PFA solution for 5 min before staining. Acridine orange staining was achieved by incubating the whole sample in an acridine orange staining solution for 45 min, prepared from acridine orange dye (Sigma-Aldrich, St. Louis, MI, USA) diluted 1000-fold in PBS. Samples were subjected to F-actin staining, and subsequently to the AcLDL uptake assay. Therefore, we used rhodamine-conjugated phalloidin dye (Abcam, Cambridge, UK) diluted 1:1000 stock solution in PBS containing 1% BSA. Nuclei were counterstained with NucBlue. CD31 staining was performed utilizing CD31/PECAM-1 mouse anti-human antibody (BBA7, R&D Systems, Minneapolis, MN, USA) in a concentration of 8 µg/mL as the primary antibody and donkey anti-rabbit Alexa Fluro 488 (Jackson ImmunoResearch, West Grove, PA, USA) at the dilution of 1:500 as the secondary antibody. Both antibodies were diluted in PBS− containing 3% donkey serum. After overnight incubation at 4 °C with the primary antibody, samples were washed with PBS−. For secondary antibody incubation, the samples were incubated for another hour at room temperature in a staining solution consisting of the secondary antibody, 1:1000 rhodamine-conjugated phalloidin dye, and NucBlue DNA staining. Finally, the samples were washed again and images were obtained with a Leica confocal imaging system (SP8) or with a conventional immunofluorescence microscope (EVOS FL Auto 2 cell imaging system, ThermoFisher Scientific, Waltham, MA, USA).

### 2.10. Data Analysis and Image Processing

Due to the low n-numbers, non-normally distributed errors can be expected for the raised data, and thus a non-parametric Kruskal–Wallis test with Dunn’s post-test was performed using GraphPad Prism version 8 for Windows, (GraphPad Software, La Jolla, CA, USA). The level of significance was set to *p* < 0.05.

Fiji 64-bit for Windows (Version 1.53c) [52] was used for image processing if not otherwise stated. For stitching the whole mount images of acridine orange-stained samples, the plugin “Stitching” of Fiji was used, based on the publication from Preibisch et al. [53], following the directions as given by the plugin description.

## 3. Results

### 3.1. Comparative Coating Efficiencies of Fibronectin-, Heparin–Chitosan- and Albumin-Coated BNC Grafts

**Figure 2:**
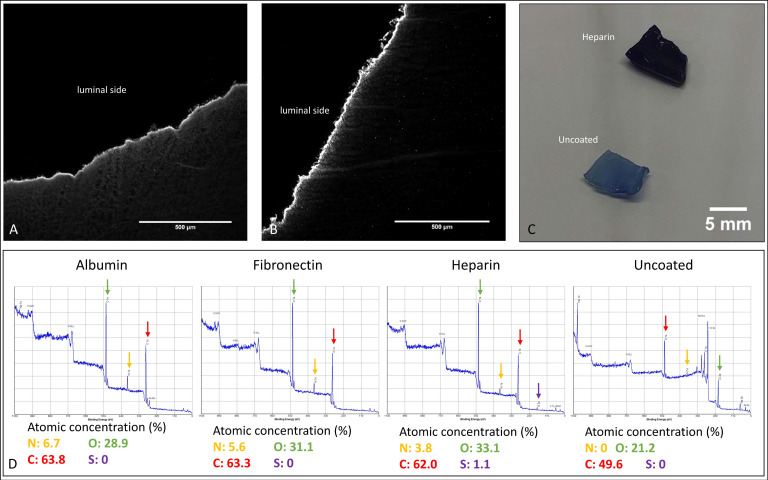
Coating analyses of exemplary coated BNC grafts: (**A**) Immunofluorescence staining of a cross-sectioned BNC graft against albumin. (**B**) Immunofluorescence staining of a cross-sectioned BNC graft against fibronectin. (**C**) Comparison of toluidine blue staining for heparin-coated and uncoated BNC grafts. (**D**) Results of XPS analyses of the coated BNC grafts. The colored arrows indicate the respective peaks for N (nitrogen), O (oxygen), C (carbon), and S (Sulphur).

In immunofluorescence images, the coated BNC grafts showed one homogenous layer of albumin and fibronectin at the luminal side of the grafts; in particular, no discontinuation of the layer was found (Figure 2A,B). Accordingly, the concentration of nitrogen at the luminal surface was clearly enhanced only for protein-coated groups, showing a concentration of 5.6% for albumin- and 6.7% for fibronectin-coated grafts on the luminal surface, as shown by XPS analyses (Figure 2D). For uncoated grafts, the concentration of nitrogen was 0%. The heparin-coated grafts stained with toluidine blue exhibited a deep blue staining covering the whole surface, compared to uncoated grafts (Figure 2C), and the detection of sulphur (1.1% vs. 0% for uncoated or albumin-/fibronectin-coated grafts) on the luminal side by XPS analyses indicated the presence of the highly sulphated glycosaminoglycan heparin. Data on the stability of the albumin and fibronectin coating are included as Table S2 in the supplementary files.

### 3.2. Progress of Metabolic Activity

**Figure 3:**
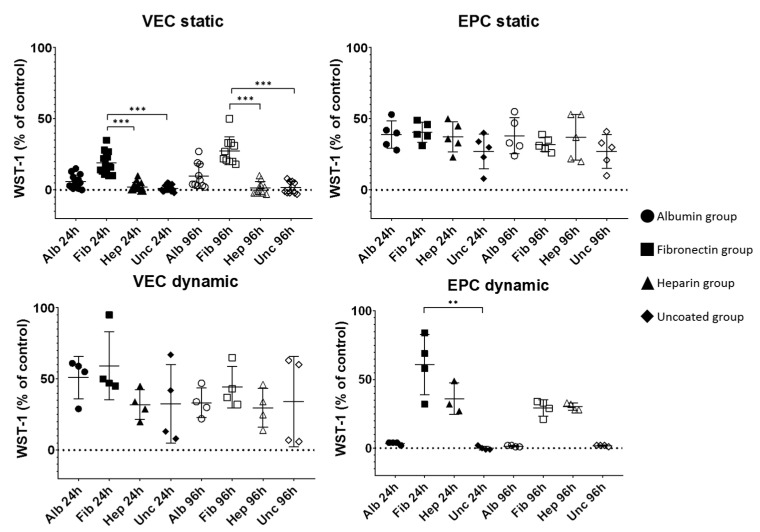
Results of WST-1 proliferation assay performed on the cell seeded BNC grafts under both static and dynamic conditions for vascular endothelial cells (VEC) and endothelial progenitor cells (EPC). The WST-1 values are normalized to control cells. *n* = 10 (VEC static), *n* = 5 (EPC static), *n* = 4 (VEC and EPC dynamic except EPC Hep 24 h (*n* = 3)). Alb—albumin, Fib—fibronectin, Hep—heparin, Unc—uncoated BNC grafts. ** *p* < 0.01, *** *p* < 0.001.

The results are summarized in Figure 3 and Table S1. In general, increased metabolic activity (as indicated by measured extinction of the WST-1 metabolite after 24 h of incubation) was considered to be increased vitalization (Figure 3). Interestingly, values clearly varied for different cell types. For static culture after 24 h, only the fibronectin group showed a recognizable vitalization by VEC cells, which revealed significantly higher metabolic activity compared to heparin-coated (18.93 ± 7.40 vs. 2.00 ± 3.12%, *p* = 0.0001) and uncoated grafts (1.07 ± 2.28%, *p* < 0.0001). After 96 h, this had not significantly changed, then showing 27.50 ± 9.74% for the fibronectin group, which was significantly higher than the heparin group (1.40 ± 4.55%, *p* < 0.0001) and uncoated group (1.70 ± 4.19%, *p* < 0.0001). The albumin group showed 6.00 ± 4.50% after 24 h and 9.70 ± 8.67% after 96 h, not reaching the level of statistical significance compared to the other groups. For dynamic cultivations, the vitalization of all groups by VEC cells had clearly increased to 51.00 ± 14.88% for albumin, 59.25 ± 23.92% for fibronectin, 32.00 ± 10.42% for heparin, and 32.50 ± 27.45% for the uncoated group, not showing statistically significant differences. After 96 h by trend, this had slightly decreased to 33.25 ± 10.44% for albumin, 44.25 ± 14.55% for fibronectin, 29.75 ± 13.57% for heparin, and 34.00 ± 31.78% for the uncoated group.

For EPC, similar values for all coatings could be observed after static culture for 24 h, where values ranged between 27.00 ± 12.29% for uncoated and 40.60 ± 7.09% the fibronectin, without reaching the level of statistical significance. After 96 h of culture, this had not changed, still showing similar values for all coatings. Dynamic culture led to clearly higher values after 24 h, but only for fibronectin (60.75 ± 21.93%) and heparin (36.00 ± 11.53%), while the others remained close to zero. After 96 h, both groups decreased to 29.25 ± 5.91% for fibronectin and 30.25 ± 2.63% for heparin, while the others still remained close to zero.

### 3.3. Histological Analyses

#### 3.3.1. Graft Colonization under Static Conditions: Acridine Orange Staining

**Figure 4:**
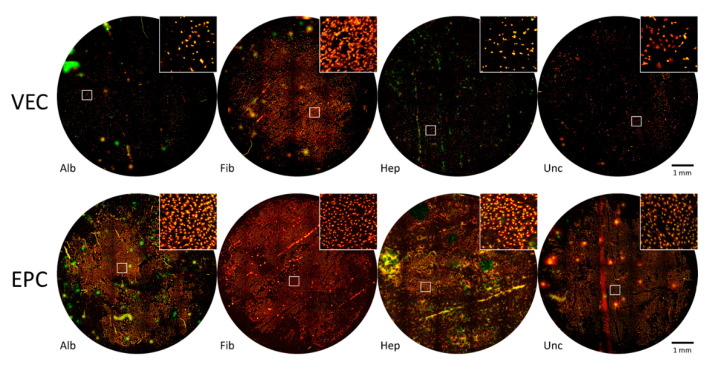
Stitched whole-mount microscopy images of acridine orange staining of BNC grafts seeded with vascular endothelial cells (VECs, upper row) and endothelial progenitor cells (EPCs, bottom row) under static conditions. The region of interest outlined in white is shown in enlarged scale in the upper right image section for each coating. While a rather confluent cell layer of VECs was only found on fibronectin-coated grafts, EPCs showed a rather confluent growth on all coatings and also uncoated grafts. Alb—albumin, Fib—fibronectin, Hep—heparin, Unc—uncoated.

Figure 4 shows whole mount images after cultivation for 96 h under static conditions, stained with acridine orange. The round image sections correspond to the total area of BNC patches seeded with endothelial cells and cultured for 96 h as described earlier.

For VECs, only fibronectin-coated patches showed multiple large and partially coherent colonized areas, but also nonpopulated spots and areas, especially close to the edge where the formerly attached pieces of PVC tube were imposed. All other coatings only showed smaller cell islets or singular cells. In contrast, for EPC, densely populated areas of EPCs were found for all coatings and also uncoated BNC patches after seeding the cells under static conditions.

#### 3.3.2. Graft Colonization under Dynamic Conditions: Acridine Orange Staining

**Figure 5:**
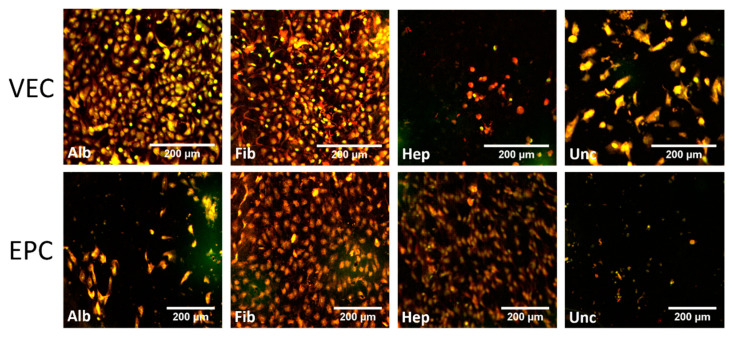
The image shows the luminal side of a sliced and spread out section of the tubular BNC graft seeded with vascular endothelial cells (VECs, upper row) and endothelial progenitor cells (EPCs, bottom row) after 96 h of dynamic cultivation, stained with acridine orange. All images reflect the exemplary regions that are labelled in Figures S3 and S4. Islands with high cell density of VECs were only found on albumin- and fibronectin-coated grafts, with lower density on uncoated grafts. The EPCs grew to highly dense populated areas only on fibronectin- and heparin-coated grafts, whereas the albumin and uncoated groups only showed isolated cells and cell groups. Alb—albumin, Fib—fibronectin, Hep—heparin, Unc—uncoated.

Under dynamic conditions, partially confluent areas of VECs were found for both the albumin and fibronectin groups, while the uncoated group showed a lower density of VECs (Figure 5 and Figure S3). The heparin group showed only scattered signals at much lower density. The most confluent coverage and highest density was clearly demonstrated by the fibronectin group.

Colonization by EPCs resulted in an apparently similar coverage for the fibronectin group (Figure 5 and Figure S4). Here, the heparin group was also colonized, but at lower cell densities. The albumin and uncoated groups remained mostly unpopulated, and only scattered cells were found.

#### 3.3.3. Cytoskeleton—Static Conditions

**Figure 6:**
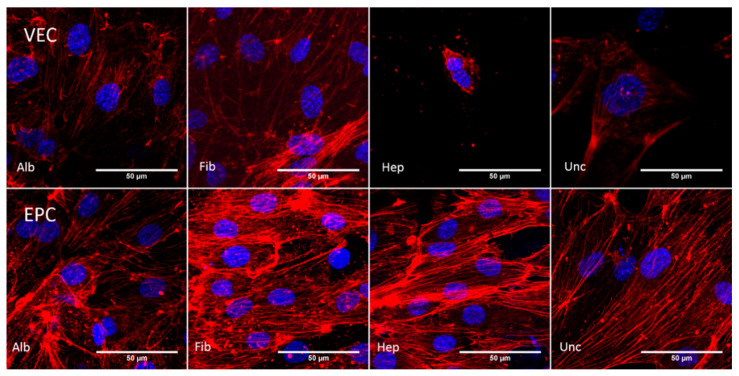
Confocal microscopy images of BNC grafts seeded with vascular endothelial cells (VEC) and endothelial progenitor cells (EPC) under static conditions. Cell nuclei were stained against DAPI (blue) and the F-actin of the cytoskeleton was stained with rhodamine phalloidin (red). All groups seeded with EPCs showed areas with dense colonization and a distinct cytoskeleton. For VECs, it was only in the heparin group that singular cells without pronounced a cytoskeleton were found, while VECs in the other groups showed areas of cells with a regular expansion of the F-actin filaments.

The F-actin staining in Figure 6 shows the cytoskeleton of the endothelial cells that were cultured on BNC patches for 96 h. For VECs, apparent stress fibers could be observed mainly in the albumin and fibronectin groups, while the heparin and uncoated groups showed rather diffuse and rare F-Actin signals, concentrated around the cell nucleus. Therefore, the albumin group showed denser and more oriented signals compared to the fibronectin group. In contrast to the VECs, apparent stress fibers could be detected in all groups colonized by EPCs. Here, F-Actin signals were generally at a higher intensity, showing denser stress fibers, which seemed to be almost equally distributed across the groups. However, the fibronectin group showed the highest density of apparent fibers, but also at the highest cell density. As apparent in the heparin group and less intensive in the fibronectin group, the fibers were more concentrated at the cell margin compared to the cytoplasmic area.

#### 3.3.4. Cytoskeleton—Dynamic Conditions

**Figure 7:**
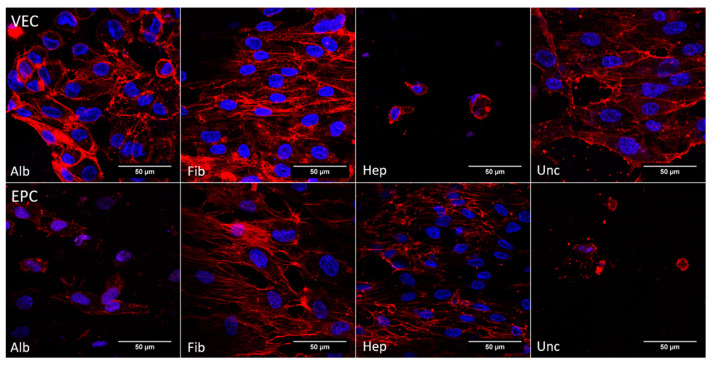
Confocal microscopy images of BNC grafts seeded with vascular endothelial cells (VEC) and endothelial progenitor cells (EPC) under dynamic conditions. Cell nuclei were stained against DAPI (blue) and the F-actin of the cytoskeleton was stained with rhodamine phalloidin (red). The fibronectin group showed a pronounced expansion of the cytoskeleton for both cell types. The VECs also showed a distinct expression of F-actin filaments on uncoated grafts. For the albumin group, the F-actin filaments were concentrated around the nuclei, indicating less spread of the cell bodies, and the heparin group showed no distinct signal of F-actin filaments. For EPCs, cells in the albumin and uncoated groups did not show clearly recognizable structures of F-actin filaments. EPCs on heparin-coated grafts showed a distinct F-actin signal.

The pattern of F-actin filaments under dynamic conditions is shown in Figure 7. Visible fibers were detected for all groups but heparin, where cells apparently were not vital. For the albumin and fibronectin groups, fibers were densely concentrated at the cell margin. While the uncoated group showed a less dense network of F-actin filaments, cells in the heparin group were only detected as scattered, singular and apparently not vital cells. This pattern of the cytoskeleton was also seen for EPCs in the uncoated and albumin groups. In contrast to this, the stress fibers of EPCs in the fibronectin group showed a strong signal, and singular fibers as well as fiber bundles at the cell margin were visible. The heparin group also showed a distinct F-actin signal, while the fibers were not visible as singular strands but rather as concentrated bundles at the cell margin.

#### 3.3.5. CD31—Dynamic Conditions

**Figure 8:**
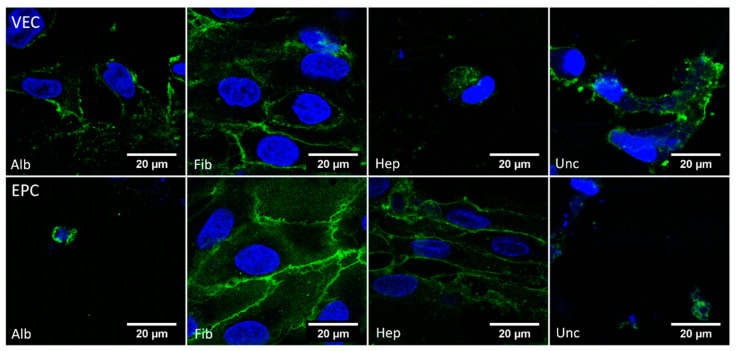
Confocal microscopy images of BNC grafts seeded with vascular endothelial cells cultivated under dynamic conditions and stained against DAPI (blue) and CD31 (green). A distinct signal of CD31 on VECs was only seen clearly in the fibronectin group, and for EPCs in the fibronectin and heparin groups.

The images obtained from parts of the BNC grafts under dynamic culture and stained against CD31 are shown in Figure 8. For VECs, a distinct signal of CD31 on the cell surface was only found for the fibronectin group, and only partially in the albumin group. In the heparin group, there were hardly any vital cells with recognizable structures and clear CD31 signals. For EPCs, a clear signal was found for the fibronectin and heparin groups, but not for the albumin and uncoated groups.

### 3.4. AcLDL Assay

#### 3.4.1. Static Conditions

**Figure 9:**
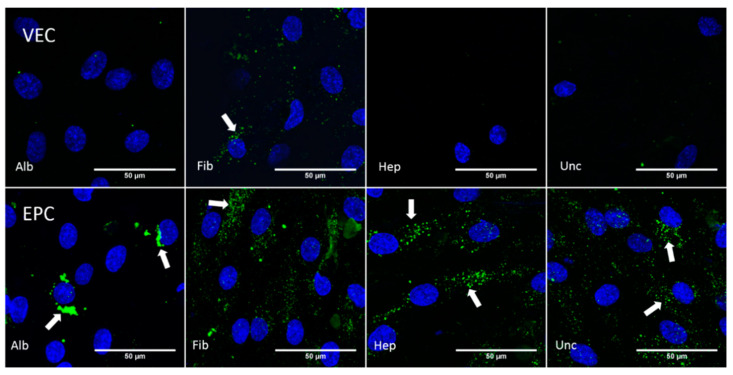
Confocal microscopy images from vascular endothelial cells (VEC) and endothelial progenitor cells (EPC) cultivated under static conditions on bacterial nanocellulose patches coated with albumin (Alb), fibronectin (Fib), heparin–chitosan (Hep) and on uncoated (Unc) patches. The cell nuclei were stained against DAPI (blue), and acetylated low density lipoprotein particles are shown in green (indicated by arrows). The VECs in the albumin and uncoated groups only showed nonspecific signals.

The results of the AcLDL uptake assay are shown in Figure 9. In the albumin, heparin, and uncoated groups, no intracellular AcLDL particles were found, while cells in the fibronectin group partially showed uptake of the AcLDL particles to the cytoplasm. In contrast to this, the EPCs showed uptake of AcLDL particles for all groups.

#### 3.4.2. Dynamic Conditions

**Figure 10:**
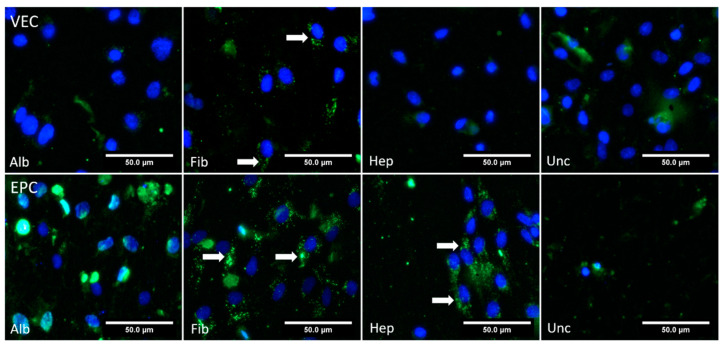
Fluorescence microscopy images from vascular endothelial cells (VEC) and endothelial progenitor cells (EPC) cultured under dynamic conditions on bacterial nanocellulose grafts coated with albumin (Alb), fibronectin (Fib), heparin–chitosan (Hep) and on uncoated (Unc) grafts. The cell nuclei were stained against DAPI (blue), and acetylated low density lipoprotein particles are shown in green (indicated by arrows).

Under dynamic conditions, partial uptake of AcLDL particles into VECs was only seen in the fibronectin group, while the other groups did not show any clear signals (Figure 10). For EPCs, intracellular AcLDL particles were present mainly for the fibronectin and heparin groups, while no specific signal was found in the albumin and uncoated groups.

## 4. Discussion

Endothelialization of small-diameter vascular grafts remains to be a crucial factor for both short- and long-term patencies, exhibited by several animal models [54,55,56,57] and also in human coronary artery bypass grafting [58,59]. This is most appealing for acellular off-the-shelf grafts, which exhibit on-site endothelialization after implantation. From the clinical perspective, the preservation of endothelial function in autologous vessels is considered to be indispensable for long-term patency [58,59]. Bacterial nanocellulose small-diameter grafts are thought to be a promising approach in the development of acellular grafts for in vivo population with ECs [14,16,17,34,60]. However, in contrast to the native extracellular matrix, BNC scaffolds fail to provide tissue-specific proteins for cell adhesion.

Accordingly, in this study, we used an uncoated BNC graft during the in vitro dynamic experimental setup, which proved to be challenging for patient-derived endothelial cells. The uncoated grafts showed poor EC growth kinetics in the WST-1 assay except for EPCs under static conditions, and only scattered cells were found, as evidenced by nucleic acids stains such as acridine orange. The morphology of EPCs was impaired as evidenced by F-actin staining, and apparently, endothelial functionality could not be preserved, as indicated by a deficiency in CD31 signals and active AcLDL uptake by both cell types. We have therefore provided an additional confirmation to show the poor performance of human microvascular ECs [61] and isolated human saphenous vein ECs [34] on uncoated grafts, which were in line with previous reports. In these studies, the authors showed that ECs seeded on unmodified BNC surfaces exhibit low adhesion and proliferation and do not spread but remain in a round shape, without the formation of a typical endothelial cell-like cytoskeleton.

Here, we contrasted the effects of coating BNC-SDVGs with different substances of previously reported relevance with regard to endothelialization in a setup using BNC-SDVGs of clinically relevant length and diameter. Thereby, we hoped to find an optimal candidate regarding adhesion and growth of clinically relevant endothelial cell types. We hypothesized that coating of small-diameter BNC grafts of clinically relevant length and diameter with one of these substances would increase the adhesion and vitality of clinical relevant EC types, such as human saphenous vein ECs (HSVECs) or EPCs. Special emphasis was given to the biological model of these two cell types, which are of particular interest because the in vivo endothelialization of vascular prosthesis depends mainly on two mechanisms: (I) the continuous ingrowth of ECs at the anastomic site, which are derived by proliferation of VECs but are often restricted to a length of 1–2 cm; (II) colonization of more distended regions by EPCs from peripheral blood [62].

Adhesion and anchorage of cells on an artificial surface is a complex process. Therefore, and though not analyzed in this study, it should also be considered that next to specific ligand binding, cell adhesion is also impacted by the biomaterial’s hydrophilicity and surface charge [63].

Regarding the surface charge, for the in vivo situation, a positive surface charge is considered to indirectly enhance cell adhesion and proliferation by enabling adsorption of plasmatic proteins, such as fibronectin, which in turn can provide specific domains for anchorage [63,64,65]. However, in vitro, it is rather the density of charges that fosters unspecific affinities to the cell adhesion molecules [66].

Unmodified BNC has been shown to have a negative charge [65], and cell adhesion could be promoted by introduction of a positive ionic charge [65,67]. Data on the surface charge of fibronectin-, albumin- or heparin–chitosan-modified BNC surfaces is limited, but fibronectin has been shown to bear a mild negative potential under experimental conditions. In contrast, albumin [68,69] and heparin [70] are both considered to be highly negatively charged molecules, while chitosan is considered to be a positively charged molecule [71,72].

As a measure for hydrophilicity, the water contact angle became a common parameter to estimate the potential for a general adhesion affinity [63]. It has been shown that the coating of biomaterials with fibronectin [21] or its binding site RGD [33], heparin [73], and albumin [27] impacts the water contact angle, where heparin- and albumin-modified surfaces showed reduced water contact angle measurements, but heparin–chitosan-modified BNC was characterized as more hydrophilic compared to native BNC [22]. Thus, regarding the expected functionality for endothelialization, the role of fibronectin as a protein of the extracellular matrix that binds via its RGD binding domain to integrins of the EC membrane (e.g., αVβ3 and αIIbβ3), allowing adhesion of the cells and thereby increasing the cell viability, is well established and widely used [35,74,75].

In contrast, mechanisms for cell adhesion on albumin seems to be rather unspecific, as integrin binding domains have not been identified for albumin yet [75]. Here, increasing the hydrophilicity of albumin-coated surfaces could improve cell adhesion, mediated by the previously mentioned indirect mechanisms by the recruitment of plasmatic proteins that are currently under debate [27,63,76].

Regarding heparin–chitosan, ECs lack a direct receptor for heparin, but it is speculated that the glucosamine group of chitosan contains domains that interact with integrins and receptors of the cell membrane, thereby modulating cell spread and proliferation [32].

After all, coating procedures for all substances could be successfully transferred to our grafts by established methodology [21,22]. After seeding and biomimetic perfusion culture in our bioreactor, the fibronectin coating clearly outperformed both albumin and heparin, as well as the uncoated BNC. Both VECs and EPCs efficiently adhered to the graft and formed densely populated colonies with preserved endothelial functions, and in addition, also built stress fibers. In the WST-1 assay, the fibronectin-coated grafts achieved approximately one third of the metabolic activity of the control groups for both cell types after a culture time of 96 h, under both static and dynamic conditions, indicating that the cells adhered well and were not washed out under flow conditions for longer hours. This was further evidenced by a dense cell population with acridine orange staining, and further, we found typical expression of stress fibers and functional integrity as evidenced by positive CD31 signals and AcLDL uptake by the cells, respectively. Importantly, this accounted for both cell types and both static and dynamic conditions, making the results most consistent compared to the other coatings, where cell adhesion and vitality differed between cell types and static or dynamic conditions.

Generally, this is in line with the established role of fibronectin as a protein of the extracellular matrix that binds via its RGD binding domain to integrins of the EC membrane, allowing adhesion of the cells and thereby increasing the cell viability [74,75]. Besides ECs, fibronectin also increases the proliferation of other SDVG-relevant cell types, such as vascular smooth muscle cells, leading to intimal hyperplasia and narrowing of the internal vessel lumen [77,78]. Previous studies with in vivo experiments have not shown excessive intimal hyperplasia in fibronectin coated SDVGs [79,80,81], but some studies have already shown that fibronectin coating increases EC adhesion to acellular vascular grafts [81,82,83,84,85] and also to BNC [21].

Kuzmenko et al. found that coating of CDAP-activated BNC with fibronectin leads to improved endothelialization with HUVECs under static conditions [21]. Recently, Osorio et al. used decellularized BNC constructs that were previously seeded with fibroblasts and showed that the BNC surface was modified with a fibronectin-containing extracellular matrix, resulting in higher cell populations in the following re-seeding experiments [86]. Consistent with the VECs that we used, Bodin et al. used HSVECs to study the repopulation of BNC modified with the fibronectin cell-binding domain RGD [33]. They found increased endothelialization for the modified BNC constructs in comparison to uncoated constructs and concluded that the RGD modification was the key factor for the improved cell adherence. We observed similar behaviors of EPCs on our fibronectin-coated grafts, which is also in line with the literature, as it is known that fibronectin coating of vascular grafts induces the homing of CD34+ endothelial progenitor cells [87] and promotes VEGF-induced CD34^+^ cell differentiation into ECs [88]. Consistent with that, our EPCs were characterized as CD34^+^ by FACS.

Importantly, it should also be considered that fibronectin coating of vascular prostheses could have unfavorable effects with regard to thrombogenicity. Fibronectin-modified surfaces are able to capture platelets via β1 and αIIbβ3 integrins, probably resulting in increased thrombogenicity [89,90,91,92], which might be counterweighted by platelet-derived EC growth factors, which in turn stimulate the growth of endothelial cells [77,93], which has already been shown by Stronck et al. with regard to endothelialization of SDVGs [94]. Also, platelet adhesion after vascular interventions is controlled by the standard administration of antiplatelet drugs [95,96], which might further lower the prothrombogenic effect of fibronectin coating.

Nevertheless, heparin and heparin–chitosan coating have also generally been shown to enhance the proliferation of ECs in vitro [22,97]. For example, Li et al. found a significant proliferation of porcine iliac ECs on heparin–chitosan-modified BNC grafts under static culture conditions [22]. Contradictorily, we found no reasonable VEC growth as the metabolic activity ranged even below uncoated BNC grafts for both static and dynamic conditions. However, the reason behind the lack of EC growth might be due to the cell type that were used in this study. Heparin has previously been shown to inhibit the proliferation of endothelial cells, particularly HSVECs [98,99,100]. Furthermore, heparin–chitosan-coated surfaces did not support the proliferation of other clinically relevant primary ECs, such as human coronary ECs [32].

Though albumin coating has been studied intensively as a candidate for the functionalization of the surfaces of medical devices in the last 30 years, data on albumin coating of vascular prostheses is limited. Importantly, it has been shown that human serum albumin inhibits apoptosis in ECs [101] and can also act as an anti-inflammatory agent by inhibiting TNFα-induced VCAM-1 expression and monocyte adhesion in human aortic ECs [102]. Furthermore, it is known that serum albumin immobilization on blood-contacting materials reduces thrombogenicity because adhesion of platelets and leukocytes is reduced [103,104]. These attributes of albumin still mark albumin as an interesting candidate for the coating of vascular prostheses.

However, in this study, VEC proliferation activity on albumin-coated grafts was only 10% of the controls under static conditions after 96 h, while up to 33% was achieved under dynamic conditions (Table S1) as measured by the WST-1 assay. Interestingly, this was flipped for EPCs, where no significant growth was seen under dynamic conditions, indicating that the cells did not adhere properly or might have been washed away during perfusion. In addition, the few adhered cells were functionally impaired, as evidenced by their inability to take up the AcLDL particles. Interestingly, in the literature, the impact of an albumin coating on endothelial cell growth is assessed ambiguously. In vivo, a bovine serum albumin aptamer coating of titanium discs, implanted in the iliac artery of dogs, enhanced proliferation of canine EPCs but not vascular ECs [27], while albumin coating of vascular Dacron prostheses did not enhance the in vivo endothelialization in another canine model [105]. In contrast to this, Krajewski et al. found that albumin-coated stents showed improved endothelialization with HUVECs [28], while others found reduced endothelialization for albumin-coated polyester grafts [29] or albumin-coated polyethylene (PET) vascular prostheses [27], when both seeded with HUVECs.

In direct comparison, EPCs exhibited improved performance for all tested parameters, even in the heparin-coated grafts. They showed reasonable growth under static conditions and were apparently not washed away under perfusion, as the metabolic activity reached approximately 30% of the controls after 96 h of dynamic cultivation. We found dense colonized areas, which is a reasonable signal of stress fibers and preserved functional integrity with respect to positive AcLDL signals.

The basic mechanism behind the different growth pattern for VECs in contrast to EPCs that were observed for the albumin- and heparin-coated grafts remains obscure. Perhaps these differences could be explained by the differences in cell proliferation potential and gene expression profile of ECs obtained from different localizations. It has been shown that EPCs provide more robust adhesion characteristics due to highly expressed genes associated with proliferation, adhesion, and motility (VEGFR1, JMJD6, and TGFB3) and indeed, they multiply rapidly in comparison to saphenous vein ECs and are more resistant to apoptosis [42,106,107,108]. So, heparin–chitosan coating still might enable colonization by EPC in vivo.

Cumulatively, the results obtained from the three different types of coated grafts reinforces the fact that except for the albumin-coated graft, where the results are still indefinite, the coating of BNC grafts with either fibronectin or heparin–chitosan remains most promising for the future development of small-diameter vascular grafts.

Taken together, this study indicates that endothelialization by the capturing of endothelial progenitors seems possible for heparin–chitosan-coated BNC grafts. Thereby, heparin–chitosan-coated grafts would be a very interesting option for small-diameter vascular grafts, even if the continuous ingrowth of ECs from the anastomic region could be impaired. It is known that intimal hyperplasia caused by smooth muscle cells at the anastomosis is one of the causes for graft failure [109,110], and interestingly, Chupa et al. found impaired proliferation and cell spread of smooth muscle cells on heparin–chitosan-modified surfaces [32], which is also confirmed by the findings of Clowes et al. [111]. Thus, a heparin–chitosan coating could probably limit the intimal hyperplasia at the anastomosis to the native vessel, while promoting endothelialization with EPCs originating from the donor blood, thereby overcoming the disadvantage of reduced attachment of HSVECs.

In summary, our data indicate that in our setting, only the groups containing integrin-binding domains—presented here by fibronectin, and potentially by chitosan—enabled colonization with preserved endothelial functionality. The albumin group revealed adhesion, but no clear signals for AcLDL uptake, and CD31 expression was low on VEC and absent on EPC.

## 5. Conclusions

In this study, we could confirm that for both VECs and EPCs, bacterial nanocellulose per se does not seem to be an attractive material for the formation of a tissue-typical endothelium. Coating with fibronectin clearly outperformed albumin and heparin–chitosan coating, regarding cell growth and preservation of endothelial functions for both cell lines. But heparin–chitosan-coated BNC grafts still allowed the attachment and spread of EPCs. Hence, for off-the-shelf small-diameter vascular grafts made of BNC, coating with either fibronectin or heparin–chitosan might promote the important endothelialization by host-cells. Further optimization is warranted and should be addressed in detail with in vivo settings.

## Data Availability

The data presented in this study are available in this manuscript and the supplementary data.

## Supplementary Materials

The following are available online at https://www.mdpi.com/article/10.3390/nano11081952/s1: Figure S1: Exemplary characterization of vascular endothelial cells (VEC) and endothelial progenitor cells (EPC) by flow cytometry. Figure S2: Exemplary characterization of vascular endothelial cells (VEC) and endothelial progenitor cells (EPC). Figure S3: Stitched microscopy images of the luminal side of a sliced and spread out section of the tubular BNC graft seeded with vascular endothelial cells after 96 h of dynamic cultivation, stained with acridine orange. Figure S4: Stitched microscopy images of the luminal side of a sliced and spread out section of the tubular BNC graft seeded with endothelial progenitor cells after 96 h of dynamic cultivation, stained with acridine orange. Table S1: Results of WST-1 proliferation assay performed on the cell seeded BNC grafts under both static and dynamic conditions for vascular endothelial cells and endothelial progenitor cells. Table S2: Protein coating stability. During the coating procedure of albumin and fibronectin patches, samples from the respective coating solution before coating and after the incubation time of 12 h were obtained, as well as from the PBS in which the samples were stored.
