# Supplementary material for: Comparative Evaluation on Impacts of Fibronectin, Heparin–Chitosan, and Albumin Coating of Bacterial Nanocellulose Small-Diameter Vascular Grafts on Endothelialization In Vitro
Source: Nanomaterials (Basel). 2021 Jul 29;11(8):1952. doi: 10.3390/nano11081952 (PMC8398117; doi:10.3390/nano11081952)
Supplement: Supplementary file 1 [file nanomaterials-11-01952-s001.zip › nanomaterials-1299057-supplementary.pdf]

nanomaterials

Supplementary Materials

Max Wacker [1,*], Jan Riedel [1], Heike Walles [2], Maximilian Scherner [1], George Awad [1], Sam Varghese [1], Sebastian Schürlein [3], Bernd Garke [4], Priya Veluswamy [1], Jens Wippermann [1] and Jörn Hülsmann [1]

[1] Department of Cardiothoracic Surgery, University Hospital Magdeburg, 39112 Magdeburg, Germany; jan.n.riedel@icloud.com (J.R.); maximilian.scherner@med.ovgu.de (M.S.); george.awad@med.ovgu.de (G.A.); sam.varghese@med.ovgu.de (S.V.); priya.veluswamy@med.ovgu.de (P.V.); jens.wippermann@med.ovgu.de (J.W.); joern.huelsmann@med.ovgu.de (J.H.)
[2] Core Facility Tissue Engineering, Otto-Von-Guericke University Magdeburg, 39106 Magdeburg, Germany; heike.walles@ovgu.de
[3] Department Tissue Engineering and Regenerative Medicine (TERM), University Hospital Würzburg, 97080 Würzburg, Germany; sebastian.schuerlein@gmail.com
[4] Institute of Experimental Physics, Otto-Von-Guericke University Magdeburg, 39106 Magdeburg, Germany; bernd.garke@ovgu.de
* Correspondence: max.wacker@med.ovgu.de; Tel.: +49-391-67-14102

1. Cell characterization by flow cytometry (FC)

The cell viability was determined using Zombie Aqua™ Fixable Viability Kit (BioLegend, San Diego, California, USA). The cell markers and the respective fluorochome used were von-Willebrand Factor (vWF)-FITC (endothelial hemostasis) (Abcam, Cambridge, UK), CD144-APC (adhesion molecule) (BioLegend), CD31-BV421 (cell adhesion molecule) (BioLegend), CD34-PE (hematopoietic origin) (BioLegend). Respective IgG controls were used to compare the expression of the markers. The intracellular staining to detect the vWF was performed using permeabilization buffer and fixation buffer (R&D Systems, Minneapolis, Minnesota, USA). Fluorescent activated cell sorting (FACS) buffer (containing PBS, 2% FBS and 2 mM EDTA) was used for the intermittent washing steps as well as for the final cell suspension before FC acquisition. The stained primary endothelial cells were acquired by BD Fortessa installed with BD FACS DivaTM software (version v9.0.1) (BD Biosciences, Franklin Lakes, NJ, USA). The acquired data were further analyzed using FlowjoTM software version 9.6.1 (Ashland, OR: Becton, Dickinson and Company, USA).

Samples from each cell pool that was used for the seeding were subjected to FC analysis, where the frozen samples were quickly thawed and resuspended in PBS. The cell numbers were adjusted to $4x10^{5}$/200 µl for FC extracellular and intracellular staining. The pelleted cells were stained for live dead stain (1:300) and incubated in dark for 20 minutes at room temperature. After washing, diluted antibody or IgG cock-tails were added to samples or control wells except vWF antibody/IgG control. Anti-Mouse Ig, κ/Negative Control Compensation Particles (BD Biosciences, Heidelberg, Germany) and VersaComp Antibody Capture Beads (Beckmann Coulter GmbH, Krefeld, Germany) were used for single stains. The cells were incubated in the dark for 30 minutes at 4 °C, followed by washing and resuspension of cells in 100 µl fixation buffer. For intracellular staining of vWF, the respective wells were washed in permeabilization buffer, and the cells were resuspended in 100 µl of vWF-FITC or IgG1-FITC and incubated for 30 minutes at 4 °C, followed by washing. The pelleted cells were resuspended in FACS buffer until acquisition.

The human saphenous vein VECs analyzed by FACs were characterized as $CD31^{high+}vWF^{high+}CD144^{+}CD34^{low+}$, and the peripheral blood EPCs were characterized as $CD31^{high+}vWF^{+}CD144^{low-}CD34^{+}$ (Figure S1). Notably, both cell types exhibited a high expression of CD31, while CD144 was detected in VECs, but not in EPCs.

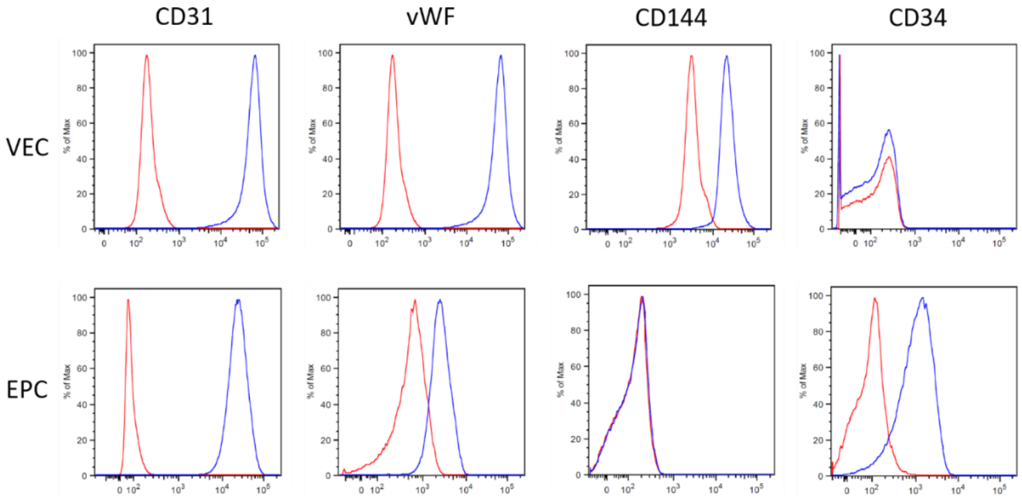

Figure S1. Exemplary characterization of vascular endothelial cells (VEC) and endothelial progenitor cells (EPC) by flow cytometry. VECs and EPCs were characterized for CD31, vWF (von Willebrand factor), CD144 and CD34 by flow cytometry, and the respective results show the expression of markers on VECs and EPCs (blue histogram) that were compared to IgG control (red histogram) as negative controls.

2. Endothelial cell culture characterization by microscopy

In phase contrast microscopy, the cells exhibited the typical endothelial cell cobblestone like morphology (Figure S2) and no contamination with fibroblasts was detected. Furthermore, a scavenger endothelial cell pathway of LDL metabolism was shown by AcLDL uptake assay (Figure S2), as indicated by the presence of intracellular fluorescence labelled AcLDL particles.

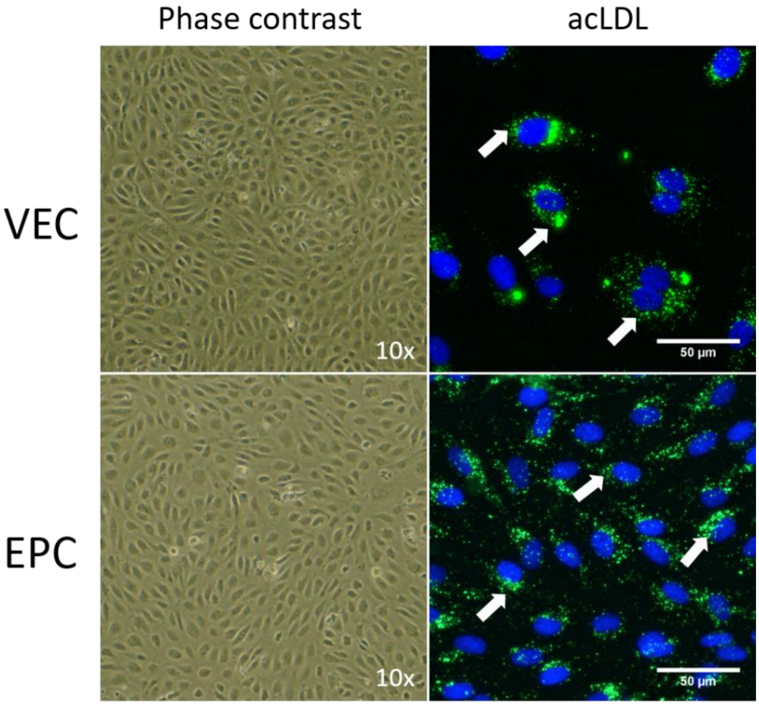

Figure S2. Exemplary characterization of vascular endothelial cells (VEC) and endothelial progenitor cells (EPC). In light microscopy images (phase contrast, left side) the endothelial cells showed a typical cobblestone like morphology. Results for AcLDL uptake assay are shown as fluorescence microscopy images (right side), where cell nuclei were counterstained with DAPI (blue) and

AcLDL particles metabolized by the endothelial cells are stained in green (arrows indicating exemplary intracellular AcLDL particles).

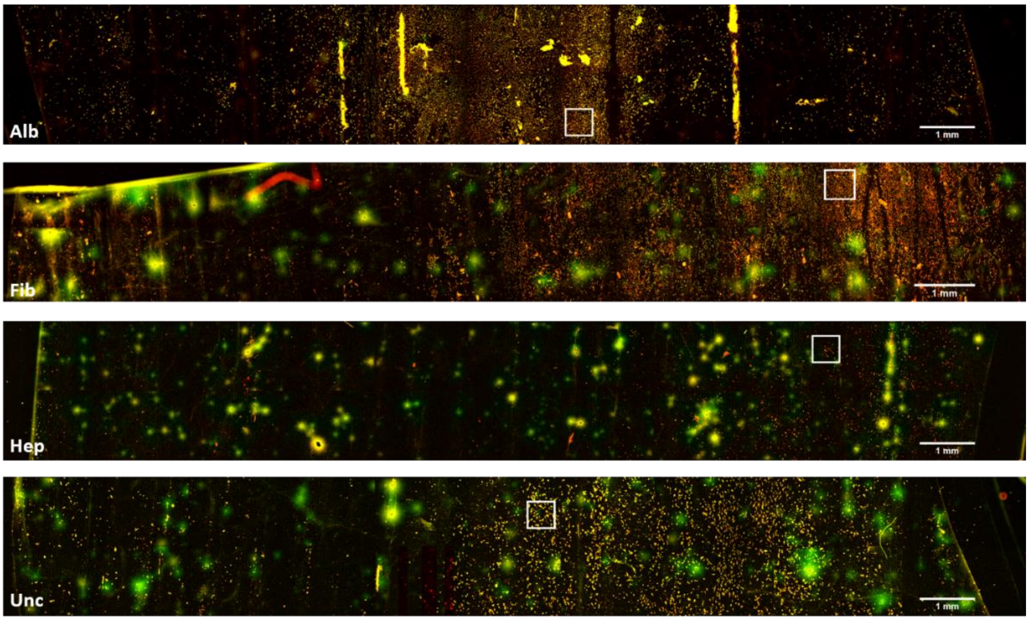

Figure S3. Stitched microscopy images of the luminal side of a sliced and spread out section of the tubular BNC graft seeded with vascular endothelial cells after 96 h of dynamic cultivation, stained with acridine orange. Exemplary regions are outlined in white and shown in enlarged scale in figure 6. Both the fibronectin and albumin group showed areas with a dense cell population, while only single cells or cell groups were found on heparin coated grafts. For uncoated grafts, several areas were populated with a less dense network of endothelial cells. Alb: albumin, Fib: fibronectin, Hep: heparin, Unc: uncoated.

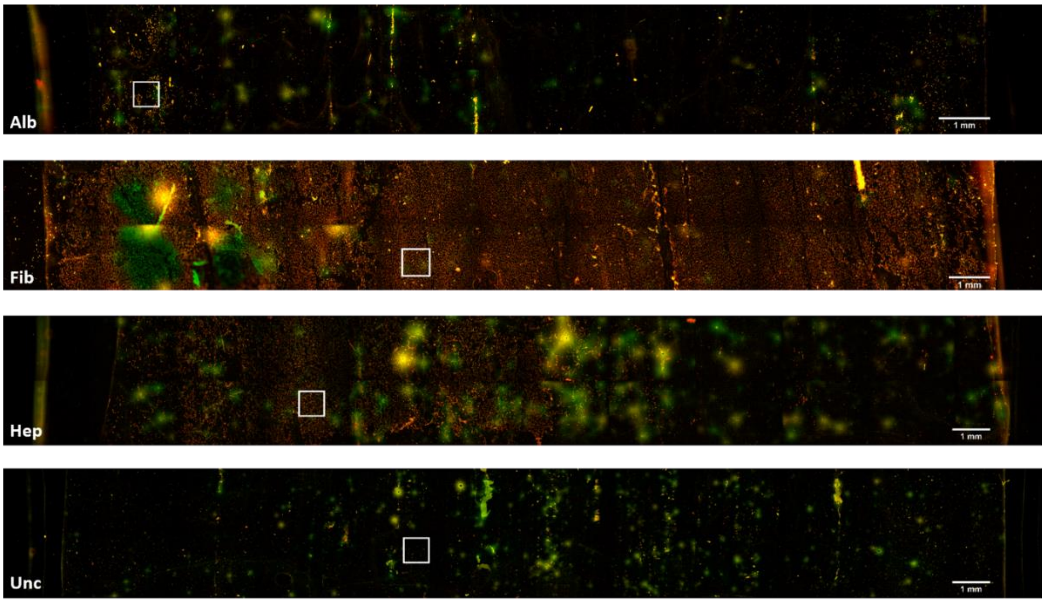

Figure S4. Stitched microscopy images of the luminal side of a sliced and spread out section of the tubular BNC graft seeded with endothelial progenitor cells after 96 h of dynamic cultivation, stained with acridine orange. Exemplary regions are outlined in white and shown in enlarged scale in figure 6. The fibronectin group showed large areas with a dense cell population, and also the heparin group showed several areas with dense population of cells, whereas in the albumin and uncoated group, only single cells or small group of cells were found. Alb: albumin, Fib: fibronectin, Hep: heparin, Unc: uncoated.

Table S1. Results of WST-1 proliferation assay performed on the cell seeded BNC grafts under both static and dynamic conditions for vascular endothelial cells and endothelial progenitor cells.

	**Alb 24h**	**Fib 24h**	**Hep 24h**	**Unc 24h**	**Alb 96h**	**Fib 96h**	**Hep 96h**	**Unc 96h**
VEC static	6.00 ± 4.50	18.93 ± 7.40	2.20 ± 3.12	1.07 ± 2.28	9.70 ± 8.67	27.50 ± 9.74	1.40 ± 4.55	1.70 ± 4.19
EPC static	39.00 ± 9.70	40.60 ± 7.09	37.40 ± 10.55	27.00 ± 12.29	38.00 ± 12.65	32.00 ± 5.01	37.00 ± 16.02	27.00 ± 11.90
VEC dynamic	51.00 ± 14.88	59.25 ± 23.92	32.00 ± 10.42	32.50 ± 27.45	33.25 ± 10.44	44.25 ± 14.55	29.75 ± 13.57	34.00 ± 31.78
EPC dynamic	3.50 ± 1.00	60.75 ± 21.93	51.75 ± 32.88	0.00 ± 1.41	1.50 ± 0.58	29.25 ± 5.91	30.25 ± 2.63	1.75 ± 0.50

Data are shown as mean % of controls ± standard deviation. Alb: albumin, Fib: fibronectin, Hep: heparin, Unc: uncoated. VEC: Vascular endothelial cells; EPC: Endothelial progenitor cells.

Table S2. Protein coating stability. During the coating procedure of albumin and fibronectin patches, samples from the respective coating solution before coating and after the incubation time of 12 h were obtained, as well as from the PBS in which the samples were stored. The total protein content in the samples (N=1) were measured in duplicates using a commercial assay (Pierce™ BCA Protein Assay Kit, catalogue number 23225, ThermoFisher Scientific, Massachusetts, USA) following the manufacturer's instructions. The results indicated that protein was successfully coated in similar concentrations on the BNC grafts and that the coating was stable with minimal release to the storage solution after 10 days.

	**Before coating**	**After 12h incubation**	**After transfer to PBS, day 0**	**Day 1**	**Day 4**	**Day 10**
**Fibronectin (µg/ml)**	118.0	6.2	0.0	9.8	0.0	3.7
**Albumin (µg/ml)**	394.8	292.3	0.0	1.3	2.4	2.3

PBS: Phosphate buffered saline.
